# Picropodophyllin causes mitotic arrest and catastrophe by depolymerizing microtubules via Insulin-like growth factor-1 receptor-independent mechanism

**DOI:** 10.18632/oncotarget.2292

**Published:** 2014-07-31

**Authors:** Ahmed Waraky, Karen Akopyan, Vendela Parrow, Thomas Strömberg, Magnus Axelson, Lars Abrahmsén, Arne Lindqvist, Olle Larsson, Eiman Aleem

**Affiliations:** ^1^ Department of Oncology-Pathology, Cancer Center Karolinska, Solna, Sweden; ^2^ Department of Cell and Molecular Biology, Karolinska Institutet, Solna, Sweden; ^3^ Axelar AB, Karolinska Institutet Science Park, Solna, Sweden; ^4^ Department of Clinical Chemistry, Karolinska Institutet, Stockholm, Sweden; ^5^ Alexandria University, Faculty of Science, Department of Zoology, Alexandria, Egypt; ^6^ The Ronald A. Matricaria Institute of Molecular Medicine at Phoenix Children's Hospital, University of Arizona College of Medicine-Phoenix, Department of Child Health, Phoenix, Arizona, USA

**Keywords:** Picropodophyllin, mitotic arrest, CDK1, IGF-1R, mitotic catastrophe

## Abstract

Picropodophyllin (PPP) is an anticancer drug undergoing clinical development in NSCLC. PPP has been shown to suppress IGF-1R signaling and to induce a G2/M cell cycle phase arrest but the exact mechanisms remain to be elucidated.

The present study identified an IGF-1-independent mechanism of PPP leading to pro-metaphase arrest. The mitotic block was induced in human cancer cell lines and in an A549 xenograft mouse but did not occur in normal hepatocytes/mouse tissues.

Cell cycle arrest by PPP occurred in vitro and in vivo accompanied by prominent CDK1 activation, and was IGF-1R-independent since it occurred also in IGF-1R-depleted and null cells. The tumor cells were not arrested in G2/M but in mitosis. Centrosome separation was prevented during mitotic entry, resulting in a monopolar mitotic spindle with subsequent prometaphase-arrest, independent of Plk1/Aurora A or Eg5, and leading to cell features of mitotic catastrophe. PPP also increased soluble tubulin and decreased spindle-associated tubulin within minutes, indicating that it interfered with microtubule dynamics.

These results provide a novel IGF-1R-independent mechanism of antitumor effects of PPP.

## INTRODUCTION

The insulin-like growth factor-1 receptor (IGF-1R) signaling pathway has been suggested as a target for cancer therapy based on studies showing that the (IGF-1R) is important for cancer cell growth and survival, and is often overexpressed in malignant [1] and premalignant tissues [2]. Inhibition of tumor growth in vivo has been achieved with anti-IGF-1R antibodies, anti-ligand antibodies, receptor-specific tyrosine kinase inhibitors, and agents such as the cyclolignan picropodophyllin (PPP) [1]. However, compared to PPP [3-7] treatment with anti-IGF-1R antibodies exhibited relatively weak anti-tumor effects in preclinical models [8, 9], and have yielded disappointing clinical data [1, 10]. Thus, raising the question whether PPP has other targets. PPP is presently undergoing clinical development in non-small cell lung cancer (NSCLC) and in glioblastoma. Tumor response was indicated in NSCLC patients included in the first clinical study, despite PPP given as third or fourth line treatment [11, 12].

Although the exact mechanism has not been established, PPP has been shown to suppress signaling in the IGF-1R pathway, reflected in enhanced degradation of the receptor and reduction of phosphorylated IGF-1R, as well as reduction of phosphorylated down-stream signaling proteins [3, 6, 13-19]. Treatment with PPP has been shown to induce apoptosis [3, 14, 15, 19-21], reduced cell motility [6, 18], and to inhibit tumor growth in xenografted mice [3, 13, 21] and in mouse tumor models [4, 5, 7]. Another characteristic outcome of treatment with PPP is the accumulation of tumor cells in the G2/M phase of the cell cycle [14, 17, 19, 20]. Interestingly, this distinguishes PPP from other molecules directly targeting the IGF-1R.

One of the key regulators of the G2/M phase transition, M-phase progression [22, 23] as well as apoptosis [24, 25] is Cyclin-dependent kinase 1 (CDK1) in complex with cyclin B1. The CDK1/cyclin B1 complex also phosphorylates and activates enzymes regulating the mitosis-specific microtubule reorganization [26-29]. It was therefore hypothesized that the activity of this complex is affected directly or indirectly by PPP. Using the distinctive PPP-induced G2/M-accumulation as a starting-point, the aim of this study was to further investigate the antitumor mechanism of action of this compound. We identified a novel, IGF-1R-independent mechanism involving an early and sustained mitotic arrest associated with pronounced activation of CDK1 in vitro and in vivo. Depending on the duration of mitotic delay, some tumor cells showed signs of mitotic catastrophe.

## RESULTS

### PPP reduced viability and induced G2/M arrest in cancer cell lines independent of IGF-1R

**Figure 1:**
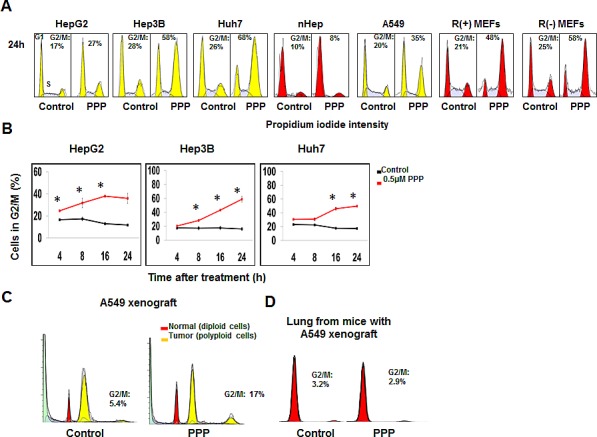
PPP induced accumulation of cancer cells in the G2/M phase A representative experiment shows cell cycle analysis using PI staining followed by flow cytometry of indicated cancer cell lines (yellow) and normal human hepatocytes, or R(+) and R(-) MEFs (red) after treatment with vehicle (control) or with PPP (0.5 μM) for 24 h (n=3) (A). The percentages of HepG2, Hep3B and Huh7 cells accumulated in G2/M phase of the cell cycle at different time points after treatment with 0.5 μM PPP are shown in (B). Results show mean ± SD, n=3, and considered significant (*) at *p* ≤ 0.05. FACS analysis of tumor (polyploid cells) and diploid cells (probably representing stromal cells) obtained from nu/nu Balb/c mice with established A549 xenografts, treated with either vehicle (left panel) or PPP (right panel) for 27 h (C). FACS analysis of murine lung tissue obtained from the same mice treated with either vehicle (left panel) or PPP (right panel) for 27 h (D). Staining of nuclei by DAPI was followed by flow cytometric analysis of cell cycle phase distribution showing G1 and G2/M peaks (yellow) for polyploid tumors and (red) for the diploid normal cells, S-phase is the hatched area between G1 and G2/M peaks (C, D). Data represent mean ± SD, n=3 (C), and n=4 (D). nHeps: normal hepatocytes, PI: Propidium iodide, PPP: picropodophyllin, R(+) MEFs: mouse embryonic fibroblasts overexpressing the IGF-1R, R(-) MEFs: mouse embryonic fibroblasts deficient for IGF-1R.

Treatment of HCC cell lines expressing different levels of IGF-1R with PPP reduced the percentage of viable cells in a dose-dependent manner (Fig. S1). These results were confirmed using the trypan blue exclusion assay. Since one of our goals was to investigate the mechanism of PPP action from a cell cycle point of view, we determined the lowest concentration (0.5 μM) that induced pronounced reduction of cell growth without massive cell death, and used this concentration for further experiments in this study. Analysis by flow cytometry showed that 24 h treatment with PPP induced G2/M phase accumulation in HCC, A549 and R+MEF (*igf1r-/-* cells overexpressing human *IGFIR*) cell lines, while normal human hepatocytes (nHeps) were not affected (Fig. 1A). The *igf1r-/-* (R-MEFs), although being deficient for the IGF-1R, also showed G2/M-accumulation in response to PPP (Fig. 1A). Kinetic studies demonstrated that the fraction of cells in the G2/M-phase increased already at 4 h and peaked between 16 and 24 h (Fig. 1B). The slight differences in response between cell lines probably reflect differences in doubling time. Similarly, data was obtained in the ex vivo analysis of A549 xenografts. PPP induced a 1.5 to 3-fold increase of tumor cells in the G2/M phase, whereas no G2/M-accumulation was observed in the normal lung tissue (Fig. 1C and D).

### CDK1 activity was upregulated in cancer cells both *in vitro* and *in vivo* after PPP treatment

**Figure 2:**
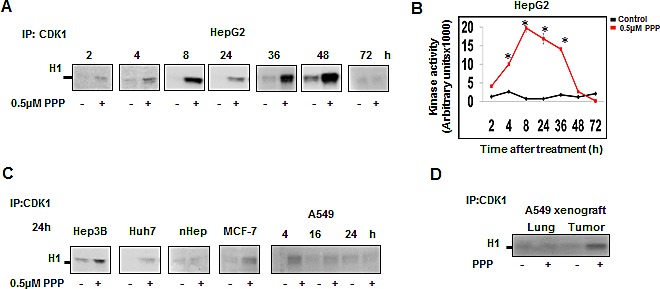
PPP induced early upregulation of CDK1 kinase activity CDK1 was immunoprecipitated using an anti-CDK1 antibody conjugated to agarose beads and the kinase activity was detected using histone H1 as substrate. HepG2 cells were treated with 0.5 μM PPP and samples taken at indicated time points (A). Quantitative analysis of the bands in (A) using image J. The red line represents CDK1 kinase activity of PPP-treated cells relative to control (DMSO) (black). Data represent mean ± SD of three experiments and considered significant (*) at *p* ≤ 0.05. (B). CDK1 kinase activity is upregulated in other cancer cell lines but not in normal hepatocytes after treatment with 0.5 μM PPP (C). CDK1 kinase activity in tumor and lung tissues obtained from A549 xenograft mice treated with vehicle or with PPP for 27 h (D). PPP: picropodophyllin, nHep: normal hepatocytes.

Since G2/M transition and M phase progression is driven by CDK1/Cyclin B, we assessed whether the PPP –induced G2/M accumulation was caused by alterations in CDK1 activity. PPP treatment was associated with CDK1 activation in all tumor cell lines (Fig. 2A, B, C) and in the A549 xenografts (Fig. 2D), whereas no CDK1 activation was detected in normal human hepatocytes or in normal lung tissue (Fig. 2C, D). CDK1 activation was evident in HepG2 cells as early as 2 h after PPP addition and persisted until 48 h. Quantitative analysis demonstrated a 2.2-fold elevation of CDK1 activity at 4 h, increasing to 21-fold at 8 h (Fig. 2A, B)

### PPP-mediated CDK1 activation was associated with an early increase in Cyclin B1 and CDK1^pT161^

**Figure 3:**
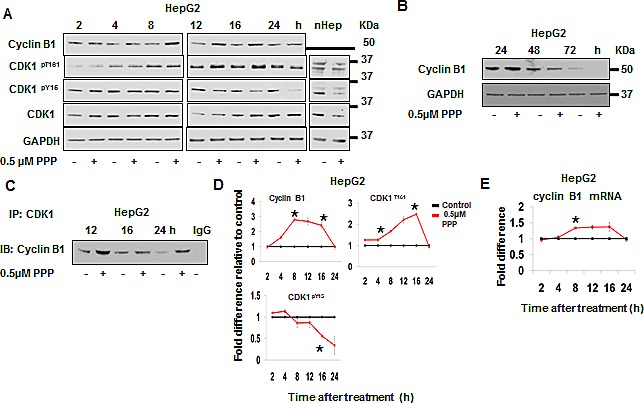
PPP induced an early increase in Cyclin B1, phosphorylation of CDK1^pT161^ and de-phosphorylation of CDK1^Y15^ HepG2 cells and nHeps were incubated with vehicle or with PPP for the indicated time points. Cell lysates were separated on 12% Bis-Tris SDS-PAGE and protein levels were detected by Western blot (15 μg protein loaded per lane). GAPDH served as a loading control (A, B). The amount of Cyclin B1 in complex with CDK1 was detected by immunoprecipitation of CDK1 followed by Western blot using a Cyclin B1-specific antibody (C). Densitometric analysis of the Western blots in (A) normalized to GAPDH are represented as fold-difference between the band intensities of PPP-treated cells (red) compared to vehicle-treated cells (black) (D). mRNA expression of cyclin B1 determined by quantitative real-time RT-PCR (TaqMan) at different time points after treatment of HepG2 cells with PPP (red) or with vehicle (DMSO) (black) normalized to GAPDH are represented as fold-difference (E). Data is presented as mean ± SD of duplicate or triplicate experiments, and considered significant (*) at *p* ≤ 0.05. nHep: normal hepatocytes, PPP: picropodophyllin.

Potential direct effects of PPP on CDK1 were analyzed in cell-free kinase assays, indicating no effects on CDK1/Cyclin A, CDK1/Cyclin E and CDK1/Cyclin B1 (data not shown). The protein levels and phosphorylation of CDK1 and its regulators following PPP treatment were studied using Western blot (Fig. 3). In comparison to control, treatment of HepG2 cells with PPP increased Cyclin B1 protein level 2.8-fold at 8 h (Fig. 3A, D), correlating with an increase in transcription (Fig. 3E). After 24 h the level of Cyclin B1 in PPP treated cells was similar to the control, and not detectable at 72 h (Fig. 3A, B, D). Similarly, the PPP-induced CDK1activity also returned to the control (untreated) levels at 72 h (Fig. 2A,B). In addition, the amount of Cyclin B1 complexed with CDK1 increased in response to PPP (Fig. 3C). The activating phosphorylation CDK1^pT161^ followed the same pattern as Cyclin B1 protein level/CDK1 activation, with a corresponding decrease in the inhibitory phosphorylation CDK1^pY15^ (Fig. 3A, D). Similar results were obtained in the A549 cell line, but with slightly different kinetics. Cyclin B1 could not be detected in the tumor tissue of the PPP-treated A549 xenografts (Fig. S2).

### PPP induced apoptosis in cancer cell lines

PPP was previously reported to induce apoptosis and CDK1 is known to regulate apoptosis. In the present study PPP induced 2.5 to 3-fold increase in apoptosis compared to controls (statistically significant only in HepG2 and MCF-7 cells) (Fig. S3A, B) with reduced levels of Mcl-1 (Fig. S3C, D). In addition, PARP cleavage was observed in MCF-7 cells after 48 h of PPP treatment (Fig. S3D). To investigate whether these alterations were due to CDK1 activity we depleted CDK1 using specific siRNA in MCF-7 cells. Depletion of CDK1 (by 80-90 %) resulted in reduced Mcl-1 levels and PARP and Caspase3 cleavage, regardless of PPP treatment (Fig. S3D).

### PPP induced mitotic arrest in cancer cell lines

**Figure 4:**
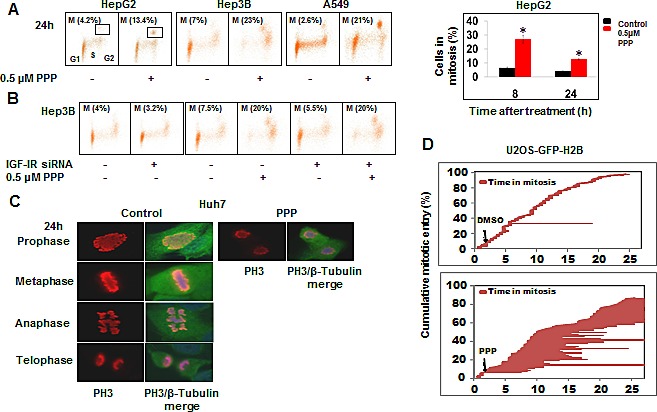
PPP induced mitotic arrest in pro-metaphase FACS analysis of HepG2, Hep3B and A549 cell lines after 24 h treatment with PPP, stained with both the mitosis marker phospho-histone 3 (ph3) and PI. A histogram showing the percentages of HepG2 cells in M-phase after 8 and 24 h treatment with vehicle (black) or with PPP (red) represented as fold-difference (A). Hep3B cells were transiently transfected with either IGF-1R siRNA or scrambled siRNA (mock) for 48h, followed by FACS analysis of transfected cells with both ph3 and PI staining (B). Dual immunofluorescence staining of ph3 (red) and tubulin (green) in Huh7 cells after treatment with PPP. The four stages of mitosis could be distinguished in the vehicle-treated cells (left panels), while the PPP-treated cells were arrested in pro-metaphase (C). Individual cell fate of U2OS cells expressing H2B-GFP was monitored by time-lapse video microscopy. Fate is depicted by horizontal lines. Each line represents a single cell with the length indicating time spent in mitosis. Cumulative mitotic entry is represented as the percentage of U2OS cells entering mitosis (y-axis) against time in hours (x-axis) for vehicle-treated cells (top panel) and PPP-treated cells (bottom panel). Arrows indicate the time point of PPP or DMSO addition (D). ph3: phospho-histone 3, PI: Propidium iodide, PPP: picropodophyllin.

Cells are expected to arrest in G2 if the CDK1/Cyclin B1 complex is inactive. However, PPP treatment yielded increased CDK1 activity. One possible explanation would be that the cell cycle arrest corresponded to accumulation of mitotic cells having high CDK1/Cyclin B1 activity. To investigate this hypothesis, arrested cells were analyzed by flow cytometry using the mitosis marker phosphorylated histone H3 (pH3). This confirmed that the cells were in fact accumulated in mitosis (Fig. 4A). In PPP-treated HepG2 cells the percentage of pH3-positive cells increased after PPP addition to 4- and 3-fold at 8 and 24h, respectively. Similar effects were observed in Hep3B and A549 cells (Fig. 4A). The potential effect of IGF-1R on the mitotic arrest was assessed in a knock-down experiment in Hep3B cells using siRNA, showing that IGF-1R depletion did not affect the PPP-induced accumulation of cells in mitosis (Fig. 4B).

Time-lapse video microscopy showed that many cells rounded up after PPP addition and contained pH3-positive condensed DNA, without signs of chromosome congression, indicating that cells were arrested in pro-metaphase (Fig. 4C). The mitotic entry/time spent in mitosis were followed for individual U2OS cells expressing GFP-Histone H2B. While control cells entered mitosis linearly, cells treated with PPP showed a minor delay in interphase (Suppl. movie 1, Fig. 4D). In contrast to control cells spending less than an hour in mitosis, mitotic progression was severely delayed immediately after the addition of PPP (Fig. 4D). This delay was pronounced also when PPP was added to cells already in mitosis (Suppl. movie 3). A fraction of PPP-treated cells eventually died, and the surviving fraction exhibited abnormal DNA content/polyploidy and enlarged nuclei, suggesting the involvement of mitotic catastrophe (Fig. S4A, B).

### PPP arrested cells in pro-metaphase by preventing the formation of a bipolar spindle and it induced spindle collapse in metaphase cells

**Figure 5:**
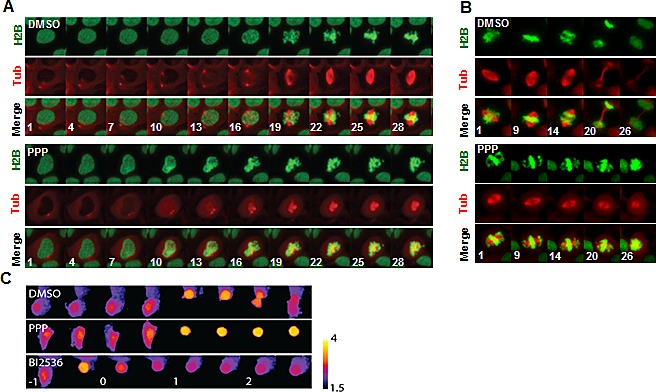
PPP interfered with mitotic spindle establishment and maintenance PPP prevented centrosome separation and establishment of a bipolar spindle during mitotic entry (A). PPP or DMSO was added to U2OS cells stably expressing GFP-Histone H2B and mCherry-a-tubulin. Cells entering mitosis were followed with time-lapse microscopy. Images show maximum intensity projections of Z-stacks with 1μM spacing. Minutes from addition of PPP are indicated. PPP disrupted mitotic spindle integrity and mitotic progression. Similar to (A) but following cells where a mitotic spindle was already established at the time of drug addition (B). PPP did not affect Plk1 activity (C). U2OS cells stably expressing a FRET-based probe to monitor Plk1 target phosphorylation were followed through mitosis. Images show false-colored inverted FRET ratio according to the displayed scale bar. The incubation time following addition of drug is indicated in hours. H2B: GFP-Histone H2B, Tub: mCherry-a-tubulin, Plk1: polo-like kinase 1, PPP: picropodophyllin.

The molecular mechanism underlying the mitotic arrest by PPP was investigated by live imaging using the U2OS cell line expressing GFP-Histone H2B and mCherry-a-tubulin. Whereas centrosome separation occurred before visible chromosome condensation in control cells, centrosomes remained close together during mitotic entry after PPP addition and a bipolar spindle never formed, leading to pro-metaphase arrest (Fig. 5A, Suppl. movie 1). No effects on centrosome maturation or the kinetics of chromosome condensation during mitotic entry were observed (Fig. 5A). When cells with established bipolar mitotic spindle were treated with PPP, the spindle collapsed and chromosome congression was reverted, showing that PPP affects both mitotic spindle assembly and maintenance (Fig. 5B, Suppl. movie 3). Bipolar spindle assembly depends on Aurora A kinase and its downstream target polo-like kinase 1 (Plk1), suggesting that the activity of these proteins might have been affected by PPP treatment. This was studied using a FRET-based sensor that monitors Plk1 phosphorylation in living cells [30]. Whereas the addition of either PPP or the Plk1 inhibitor BI2536 caused arrest in pro-metaphase, only BI2536 decreased the inverted FRET-ratio in both G2 and mitosis. This indicated that PPP did not induce a mitotic arrest by the inhibition of Aurora A or Plk1 kinase activities (Fig. 5C, Suppl. movie 2).

**Figure 6:**
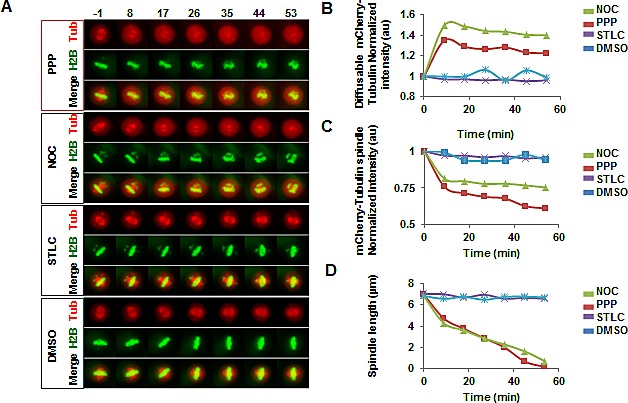
PPP addition in metaphase led to spindle collapse, increased levels of diffusible mCherry-a-tubulin, and decreased levels of spindle-associated mCherry-a-tubulin U2OS cells stably expressing GFP-Histone H2B (H2B) and mCherry-a-tubulin (Tub) were blocked in metaphase by treatment with 20 μM MG132 for 1.5h, followed by time-lapse microscopy. After acquisition of the first image, 0.5 μM PPP, 25 ng/mL Nocodazole (NOC), 40 μM S-Trityl-L-Cysteine (STLC), or DMSO were added (A). Images show maximum intensity projections of mCherry-a-tubulin, GFP-Histone H2B, and the merged channels (Merge). Time (minutes) from addition is indicated above images. Top graph shows normalized quantifications on average mCherry fluorescence in area of the cell where no visible spindle structure is present, indicating the free pool of diffusible mCherry-a-tubulin (B). Middle graph shows normalized quantifications of maximum mCherry intensity in cells, indicating accumulation of mCherry-a-tubulin in mitotic spindle (C). Lower graph shows distance between centrosomes (D). All values are averages of at least 7 cells per condition.

Addition of PPP in metaphase led to a gradual decrease in spindle length that eventually resulted in bipolar spindle collapse (Fig. 6A, D, Suppl. movie 3). Centrosome separation and bipolar spindle assembly mechanistically depend on the motor protein kinesin-5 (Eg5) [31]. However, Eg5 is not essential for maintenance of a bipolar spindle when cells are arrested in metaphase [22, 23]. Thus, although addition of the Eg5 inhibitor STLC before mitosis led to inhibition of centrosome separation and pro-metaphase arrest (data not shown), addition of STLC to cells that were arrested in metaphase by the proteasome inhibitor MG132 did not lead to a mitotic spindle collapse (Fig. 6A, D, Suppl. movie 3). In contrast, addition of PPP in metaphase led to a gradual decrease in spindle length that eventually resulted in bipolar spindle collapse, indicating that PPP does not function through inhibition of Eg5 (Fig. 6A, D, Suppl. movie 3).

### PPP interfered with microtubule dynamics

The effects of PPP on cells arrested in metaphase by MG132 were compared with the effects of the microtubule destabilizer Nocodazole (NOC), and the Eg5 inhibitor STLC. Treatment with PPP or NOC led to a collapse of the mitotic spindle, whereas STLC did not have any detectable effect (Fig. 6A, D, Suppl. movie 3). Importantly, whereas mCherry-a-tubulin distribution remained constant after STLC or DMSO addition, the addition of either PPP or NOC in the same phase of cell division led to an increase in the pool of mCherry-a-tubulin outside of the spindle (Fig. 6B, Suppl. movie 3), and to a decrease in the amount of mCherry-a-tubulin associated with the spindle (Fig. 6C, Suppl. movie 3). This phenotype was evident in all 26 PPP-treated cells studied in the experiment.

**Figure 7:**
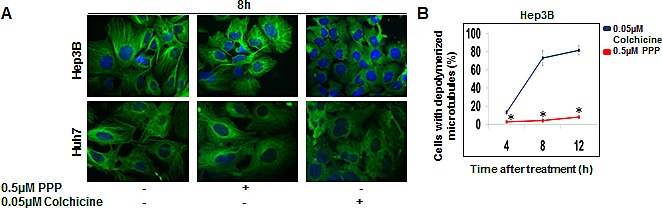
Effect of PPP on microtubule organization in interphase Hep3B and Huh7 cells were treated with either 0.5 μM PPP or 0.05 μM colchicine, followed by visualization of b-tubulin by immunofluorescence using an Alexa-conjugated b-tubulin binding antibody. After 8 h treatment microtubule organization appeared intact in vehicle-treated control cells (DMSO) (left panels) and in PPP-treated cells (middle panels). Microtubule disorganization was observed following treatment with 0.05 μM colchicine (right panels) (A). The fraction of cells with disorganized microtubules at different time points were calculated and depicted in a histogram (B). Data represent mean ± SD, n=3, and considered significant at *p* ≤ 0.05. (*) both between colchicine- and PPP-treated cells and between PPP- and vehicle-treated cells.

The potential effects of PPP on microtubule dynamics during interphase were compared with the effects of the microtubule depolymerizer colchicine. The number of cells without visible microtubules was counted using immunofluorescence microscopy. A substantial effect was observed 8 h after colchicine addition, whereas PPP had minor effects (Fig. 7A, B). The effect seen with PPP at 12 h did reach statistical significance compared to the starting value for PPP treated cells, but remained below the starting value for the colchicine treated cells.

Several experiments were performed to investigate whether PPP might bind directly to microtubules. We measured displacement of tritiated colchicine, PPP, or PPT (Fig. S5). Colchicine and PPT competed for binding to b-tubulin, whereas PPP did not displace colchicine, despite that a concentration as high as 50 μM was used. For PPT, this displacement resembled the pattern for cold colchicine (Fig. S5). In a final competition experiment, no binding curve for tritiated PPP binding to b-tubulin could be established. The weak signal could not be blocked with cold PPP (or cold PPT) confirming that this was not a result from specific binding. Thus, PPP mediates a mitotic arrest by interfering with microtubule dynamics, without binding to b-tubulin.

## DISCUSSION

In this study we confirmed that the PPP-induced G2/M arrest is independent of IGF-1R because it occurred in *igf1r*-/- (R-) MEFs and after IGF-1R downregulation using siRNA in cancer cell lines. The G2/M arrest also occurred in cancer xenografts in vivo after PPP treatment.

Detailed studies showed that the PPP-induced G2/M arrest paralleled the activation of Cyclin B1/CDK1 activity in vitro and in vivo, indicating that cells accumulated in mitosis. This was confirmed by phospho-histone H3 positive staining of the growth arrested tumor cells. Therefore, the “G2/M” arrest observed in the propidium iodide-based FACS analyses in this and in previous reports is in fact a mitotic arrest.

Real-time imaging of a U2OS cell line stably expressing GFP-Histone H2B and mCherry-a-tubulin confirmed that the cells were arrested in mitosis. Addition of PPP before mitotic entry inhibited centrosome separation and the formation of a bipolar mitotic spindle, leading to pro-metaphase arrest. PPP addition to cells already in mitosis led to a collapse of the mitotic spindle. Changes in mitotic spindle structure occurred within 10 minutes after addition of PPP, indicating that PPP acts directly on a regulator of the mitotic spindle and not through effects on transcription or protein synthesis. The effect after addition of PPP during metaphase was shown to be similar to the effect seen after addition of the microtubule destabilizer nocodazole (NOC). In both cases collapse of the mitotic spindle was observed, associated with decrease in the amount of tubulin associated with the spindle and concomitant increase in the tubulin pool outside of the spindle.

Cells cannot remain permanently arrested in pro-metaphase [32]. In the present study some of the mitotically arrested cells were found to die in mitosis and the remaining cells exhibited enlarged polyploid nuclei, a scenario similar to that described for mitotic catastrophe [33, 34]. Indeed, cells may survive mitotic arrest by exiting mitosis without completing cell division (mitotic slippage) to become multinucleated [34]. It has been proposed that the inhibition of transcription during mitotic arrest and its restoration after mitotic slippage explain the different scenarios of cell fate determination after mitotic arrest [34]. Inhibition of transcription during mitotic arrest mediated by PPP might explain the reduced levels of the major microtubule-associated protein Tau in PPP-treated A549 xenografts observed in the present study (data not shown). This notion is corroborated by the fact that reduction in Tau was not observed in normal lung tissue from the same mice, because lung tissue is quiescent. Furthermore, mitotic arrest occurred within minutes in the A549 cell line but Tau reduction was observed in the same cell line after 16 h.

It was recently reported that exposure to PPP, at a very high concentration (i.e. 10 μM), has an effect on microtubules, leading to blurry tubular staining in micrographs taken after 24 or 48 hours [35]. The authors suggested that this effect is due to PPP binding weakly to b-tubulin at the same location as colchicine and the PPP diastereomer podophyllotoxin (PPT). However, binding competition studies using tritiated colchicine as the tracer have previously shown that PPP does not compete with colchicine, in contrast to PPT [36]. This experiment was extended in the current study, measuring displacement of tritiated colchicine, PPP, or PPT, by the unlabeled compounds. In agreement with previous data colchicine and PPT competed for binding to b-tubulin, whereas PPP did not. Furthermore, in the third part of the experiment no specific binding of tritiated PPP to b-tubulin could be established.

Centrosome separation and the formation of a bipolar mitotic spindle requires controlled changes in microtubule dynamics [37]. These processes depend on the activities of Aurora A kinase and its downstream target Plk1, which has prompted interest in their function as anti-cancer targets [38]. However, the present study showed that Plk1 activity remained high after addition of PPP, indicating that PPP does not function through Plk1. The study also showed that PPP does not function through inhibition of the motor protein Eg5, needed for centrosome separation [31].

To investigate whether PPP could induce rapid effects on the microtubule system in other cancer cells in interphase immunofluorescence microscopy was used, and we could only detect weak depolymerizing effects in comparison to a very low dose of the microtubule inhibitor colchicine that induced drastic depolymerisation of the microtubule system. This together with previous results indicated that PPP has only a very weak microtubule inhibitor activity in this experimental setting.

A number of microtubule inhibitors are presently used in clinical oncology. They have severe adverse effects including bone marrow depression, gastrointestinal and neurological disturbances [39]. In contrast, PPP has been shown to be well tolerated in patients despite administration twice daily for weeks and months, with reversible neutropenia as the only dose-related and dose-limiting toxicity [11]. The chief limitation of microtubule-targeted agents is the high rate of neuropathy induced by these agents [40]. The gastrointestinal side effects of microtubule-inhibiting drugs, i.e. diarrhea, melena and paralytic ileus, are probably explained by the high proliferative activity in the intestinal mucosa. No drug-related, clinically important gastrointestinal or neurological events have been reported after oral administration of PPP in the clinic [11, 12]. These early clinical data indicate that PPP does not show the typical side effects of general microtubule inhibitors thus suggesting that PPP might affect microtubule dynamics in a different manner than currently available drugs. Microtubule dynamics is spatially highly regulated in mitosis [26, 37], and it is possible that PPP affects proteins that regulate this process. Furthermore, binding to polymerized or modified forms of b-tubulin found in the cellular context cannot be ruled out.

In the present study PPP treatment resulted in prominent CDK1 activation with increased levels of Cyclin B1 protein and mRNA, increased Cyclin B1 in complex with CDK1, and CDK1^Thr161^ phosphorylation in cancer cell lines or tumors. PPP did not seem to directly target CDK1 and the activation of CDK1 by PPP in cell lines occurred the earliest after 2-4 h incubation, while the interference with tubulin and spindle formation occurred after minutes of PPP addition. Therefore, the CDK1 activation induced by PPP may be a consequence of its effect on mitosis. Other compounds affecting the microtubules have been shown to induce similar effects [41, 42]. PPP did not induce G2/M arrest or CDK1 activation in normal human hepatocytes (nHeps) or in normal lung tissue from xenografted mice. This may be explained by the fact that nHeps and cells in lung tissue are quiescent and do not enter mitosis [43]. In agreement, we further investigated the effect of PPP on the proliferating normal human fibroblast cell line GM2808. We observed a 1.5-fold increase in the percentage of cells in the G2/M phase of the cell cycle after 24 h treatment with the same dose of PPP (data not shown). This supports the fact that PPP selectively eliminates proliferating cells, which is relevant for cancer therapy, aiming to target the proliferating cancer cell that invades and metastasizes [44].

In the present study PPP induced statistically significant apoptosis only in HepG2 and MCF-7 cell lines and not in Hep3B or Huh7 cells. This was accompanied with a reduction in Mcl-1. The reduced Mcl-1 levels may be due to its degradation by CDK1 phosphorylation because CDK1 knockdown using siRNA rescued these effects. This is in agreement with previous results [24]. HepG2 and MCF-7 cell lines have wild type p53, whereas Hep3B lacks p53 and Huh7 harbors mutated p53 [45]. During PPP-induced mitotic arrest, mitotic inhibition of transcription may cause the level of Mdm2 mRNA and other short-lived mRNAs to decrease rapidly. Subsequent loss of Mdm2 results in stabilization of p53 in cell lines having wild type p53. Furthermore, due to more long-lived p53 mRNA synthesis may continue for some time after a block in transcription, causing p53-dependent apoptosis (reviewed in [34]). A recent study supports that the p53-status may explain the sensitivity to PPP [46].

In conclusion, our results demonstrate a novel mechanism of action of the anticancer agent PPP, interfering with microtubule dynamics and leading to mitotic arrest. The effect is observed within minutes, and is not caused by direct effects of PPP on b-tubulin, CyclinB/CDK1, Aurora A, Plk1, Eg5. We suggest that the microtubule effect is mechanistically separate from the widely reported effects on IGF-1R pathway. It is likely that the ability to cause mitotic arrest of tumor cells is an essential component of the anti-tumor efficacy of PPP, since this may lead to mitotic catastrophe and subsequent cell death.

## MATERIALS AND METHODS

### Cell lines and reagents

HepG2 cell line (DSMZ, Braunschweig, Germany) was cultured in RPMI 1640 (Gibco, Paisley, UK) with 10% fetal bovine serum (FBS; Hyclone, Logan, UT), whereas Hep3B (ATCC, Manassas, VA) and Huh7 (Professor M. Ingelman-Sundberg, Karolinska Institutet, Stockholm, Sweden) were cultured in DMEM (Gibco) with 10% FBS. As a normal counterpart to these hepatoma cell lines, human hepatocytes (nHep) (Lonza, Basel, Switzerland) were used and maintained in hepatocyte basal medium supplemented with the HCM™ bullet kit^®^ (Lonza). A549, MCF-7 (Professor K. Wiman, Karolinska Institutet), *igf1r-/-* mouse embryonic fibroblasts (R-MEF), IGF-1R-overexpressing (R+MEF) (Professor R. Baserga, Thomas Jefferson University, PA) and U2OS expressing green fluorescent protein (GFP)-Histone H2B (J. Raaijmakers and R. Medema, Dutch Cancer Institute, the Netherlands) were cultured in DMEM with 10% or 6% FBS (U2OS). All cell lines were maintained in complete medium with penicillin (100 units/ml), streptomycin (50μg/ml) (Hyclone, Logan, UT) at 37°C in a humidified atmosphere containing 5% CO_2_ and confirmed authentic using Short Tandem Repeat kit AmpFSTR identifier (Applied Biosystems, Foster City, CA). PPP and picropodophyllotoxin (PPT) were synthesized as described [3], and similar to colchicine, nocodazole, MG-132 (Sigma, St Louis, MO), S-Trityl-L-Cysteine (STLC) (Tocris Bioscience, Ellisville, MO) and BI 2536 (Selleckchem, Houston, TX) were dissolved in dimethylsulphoxide (DMSO).

### A549 xenograft model

A549 cells (2x10^6^) in sterile phosphate-buffered saline (PBS) were injected subcutaneously in the ﬂanks of 5-6 week-old immunodeﬁcient, female nu/nu Balb/c mice. After 50 days, mice were treated using an oral gavage with either PPP (40 mg/kg) or vehicle (7.5 mL/kg) at 0, 12 and 24 h with daily monitoring of tumor growth using a vernier caliper. At 3 h after the last treatment, animals were sacrificed, tumor weights and volumes determined and tumor and lung tissues were collected, snap-frozen in liquid nitrogen and stored at -80°C. Animal experiments were performed by ProQinase (Freiburg, Germany) according to the guidelines of the Federation for Laboratory Animal Science Associations, EU.

### Analysis of cell growth, cell cycle distribution and apoptosis

Cell viability was determined with the alamarBlue® assay (Invitrogen) as described in [47]. Cell cycle phase distribution was analyzed by flow cytometry using propidium iodide (PI) as described in [14]. For M-phase quantification, cells were fixed with 70% ethanol, permeabilized using 0.25% TritonX-100, incubated with anti-phospho-histone H3 (pH3) antibody 06-570 (Millipore, Temecula, CA) for 1 h at room temperature (RT), washed with PBS containing 1% bovine serum albumin (BSA) and incubated with goat anti-mouse Alexa Fluor 488-conjugated antibody A11029 (Invitrogen) and PI. Following analysis with flow cytometry, the data were processed with the Cell Quest software (Becton Dickinson, San Jose, CA) or ModFit Software (Verity Software, Topsham, ME). For the analysis of cell cycle phase distribution of cells from tumor and lung tissue of A549 xenografts, the formalin-protease method was adopted as described [48], followed by quantification using ModFit Software. Apoptosis was studied using Annexin V/PI method (Annexin V-FLUOS staining kit, Roche, Mannheim, Germany) according to the manufacturer's protocol. Flow cytometric analysis was immediately performed using FACS Calibur.

### siRNA knockdown of IGF-1R and CDK1

IGF-1R- and mock-siRNA (*Thermo Fisher* Scientific, Waltham, MA) were transfected into Hep3B cells at 40% confluence by adding 8 μL Dharmafect 4 (Invitrogen, Carlsbad, CA) and 40 nmol siRNA to 2 mL antibiotic-free medium. CDK1- and mock-siRNA (Qiagen, Hilden, Germany) were transfected into MCF-7 cells by adding 12 μL of oligofectamine (Invitrogen, Carlsbad, CA) and 10 nmol of siRNA to 2 mL antibiotic-free medium. After 48 h (IGF-1R siRNA) or 72 h (CDK1 siRNA) the cells were washed with fresh medium and then treated with PPP or vehicle for an additional 24 h before analysis. The knockdown efficiency was 80-90% as assessed by Western blotting.

### Western blotting

Cells were lysed in modified RIPA buffer (50 mM Tris pH 7.4, 150 mM NaCl, 1% NP-40, 1 mM EDTA, 0.25% sodium deoxycholate) containing protease (Roche) and phosphatase (Sigma) inhibitors. Lysates were centrifuged at 16,100 x *g* at 4ºC for 30 min and supernatants stored at -80ºC. Protein concentrations were determined using BCA (Pierce Biotechnology, Rockford, IL) and equal amounts of protein were resolved on 4-12% gradient- or 12% Bis-Tris SDS-PAGE gels (Invitrogen), transferred to nitrocellulose Hybond membranes (Amersham, *Buckinghamshire, UK*), blocked with 5% dry milk or BSA in 20 mM Tris base, 135 mM NaCl, 0.1% Tween 20 (TBST) and blotted against rabbit anti-caspase3 #9662, rabbit anti-CDK1^pT161^ #9114, rabbit anti-CDK1^pY15^ #9111, mouse anti-Cyclin B1 #4135, and rabbit anti-Mcl-1 #5453 antibodies (Cell Signaling Technology, Beverly, MA) or rabbit anti-GAPDH sc-25778 antibody (Santa Cruz Biotechnology, Santa Cruz, CA) or mouse anti-PARP #556363 (Becton Dickinson) or rabbit anti-CDK1 #PC25 antibody (Calbiochem, San Diego, CA). Membranes were routinely washed using TBST, and incubated with secondary anti-rabbit/mouse/goat IgG horseradish peroxidase-conjugated antibodies NA934, NA931 (Amersham) or 31402 (Pierce Biotechnology) followed by signal detection using enhanced luminescence Hyperfilm-ECL (Amersham) and quantification using imageJ.

### CDK1 kinase assay

Cells/tumor tissue were lysed in PBS pH 7.0 containing 10% glycerol, 0.5 mM EDTA, 1 mM DTT, 2 mM NaF, 0.2% Triton-X 100, protease/phosphatase inhibitors [47] and processed as described above. CDK1 was immunoprecipitated using mouse anti-CDK1 agarose-conjugated antibody sc-54 (Santa Cruz Biotechnology) followed by washing and re-suspension in kinase buffer with 10 mM DTT and 20-50 μM unlabeled ATP. After addition of 1.5 g histone H1 (Roche) and 5-10 μCi γ-^32^P (Perkin Elmer, Rodgau, Germany), the samples were incubated for 30 min at 30°C with termination by 4 x SDS-PAGE sample buffer followed by electrophoresis on 12% polyacrylamide gel with final analysis/quantification by autoradiography/densitometry [47].

### RNA isolation and real-time RT-PCR

Total RNA was isolated using the RNeasy kit (Qiagen) and stored at -80°C until use. The concentration and the quality of isolated RNA were determined using NanoDrop1000 spectrophotometer (NanoDrop Technologies, Wilmington, DE). cDNA was generated from 2 μg of total RNA with Reverse Transcription Kit (Applied Biosystems) and the concentration determined as above. Real-time RT-PCR was carried out (10 min 95ºC, 40 cycles 15 s 95ºC, 1 min 60ºC) using the ABI 7500 probes for cyclin B1 (Hs00259126_m1) and GAPDH (Hs02758991_g1), each having the fluorochrome FAM conjugated at the 5' end (Applied Biosystems).

### Immunofluorescence analysis of tubulin and phosphohistone H3 (pH3)

The cells, seeded on coverslips, were fixed in 4% buffered paraformaldehyde solution, permeabilised using 0.1% TritonX-100, incubated overnight with rabbit anti-pH3 antibody 04-817 (Millipore), washed with PBS containing 5 mM EGTA and incubated with goat anti-rabbit Alexa Fluor 594-conjugated antibody A11012 (Invitrogen) for 1 h at RT. For subsequent staining of tubulin, cells were washed with PBS-EGTA, incubated with mouse anti-tubulin antibody T5168 (Sigma) for 1 h at RT, washed with PBS-EGTA and incubated with goat anti-mouse Alexa Fluor 488-conjugated antibody A11029 (Invitrogen). For counterstaining of cell nuclei, the coverslips were mounted using Vectashield mounting medium containing DAPI (*Vector *Laboratories, Burlingame, CA). Cells were examined using an Axioplan 2 fluorescence microscope (Zeiss, Jena, Germany).

### Live cell imaging

Time-lapse video microscopy was performed at 37°C with 5% CO_2_ on an inverted Leica DMI6000 microscope using NA 0.4 and 0.85 dry objectives. Images were acquired with a Photometrics Evolve 512 EM-CCD camera using Leica Application Suite software and analyzed by using ImageJ. For images of U2OS cells expressing both mCherry-a-tubulin and GFP-Histone H2B, Z-stacks with a spacing of 1 μm were acquired and are represented as maximum intensity projections. mCherry-a-tubulin association to the mitotic spindle was measured by taking the maximum mCherry intensity of the cell, whereas the diffusible pool of mCherry-a-tubulin was assessed by measuring the average mCherry fluorescence in an area of the cell where no visible spindle structure is present. Inverted fluorescence resonance energy transfer (FRET) ratios of a Plk1-responsive probe [30] were monitored and analyzed as described [49].

### Tubulin-binding assay

Binding of PPP and PPT (both from the Biovitrum compound collection) and colchicine (Sigma C-9754) were performed using Colchicine site competitive assay kit Cytodynamix Screen 15 (Cytoskeleton, CDS15), according to the manufacturer's instruction. In separate experiments [^3^H]-Colchicine (American Radiolabeled Chemicals, ART-722), [^3^H]-PPP (Biovitrum) or [^3^H]-PPT (Biovitrum) were used as tracers, having specific activities of approximately 3145, 670, and 670 GBq/mmol, respectively. The concentration of [^3^H]-colchicine in the assay was 53 nM, and the concentration of [^3^H]-PPP or [^3^H]-PPT was 4-fold higher, due to lower specific activity, keeping within the limit of the kit procedure as stated by the manufacturer. Compound stock solutions were 10mM in DMSO, and serial dilutions 1:3 were made in DMSO prior to transfer to the assay plate. Top concentration of compound dilution in the assay was 42 μM. Serial dilutions were made in duplicates. Briefly, 10 μL of compound dilution and 10 μL of tracer dilution were incubated with 180 μL of tubulin-biotin SPA-beads for 45 minutes at 37 °C after a short initial shake. Readings were done using a 1450 MicroBeta Trilux 32 BI. Data were analysed using Xlfit 4 parametric linear regression model 205.

### Statistical analysis

Statistical significance was defined as *p* ≤ 0.05 based on two-sided Student's t-test.

## SUPPLEMENTARY MATERIAL AND FIGURES


