# Supplementary material for: Picropodophyllin causes mitotic arrest and catastrophe by depolymerizing microtubules via Insulin-like growth factor-1 receptor-independent mechanism
Source: Oncotarget. 2014 Jul 31;5(18):8379–92. doi: 10.18632/oncotarget.2292 (PMC4226690; doi:10.18632/oncotarget.2292)
Supplement: Supplementary file 1 [file oncotarget-05-8379-s001.pdf]

Picropodophyllin causes mitotic arrest and catastrophe by depolymerizing microtubules via Insulin-like growth factor-1 receptor-independent mechanism

Supplementary Material

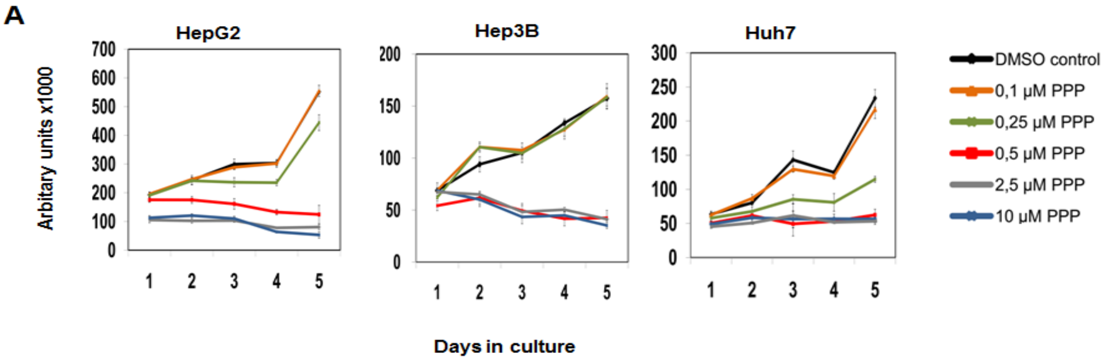

Supplementary figure 1: PPP reduced the viability of liver cancer cell lines in a dose-dependent manner. HepG2, Hep3B and Huh7 cells were treated with various concentrations of PPP for 5 days. Cell viability was assessed by Alamar blue® fluorescence assay.

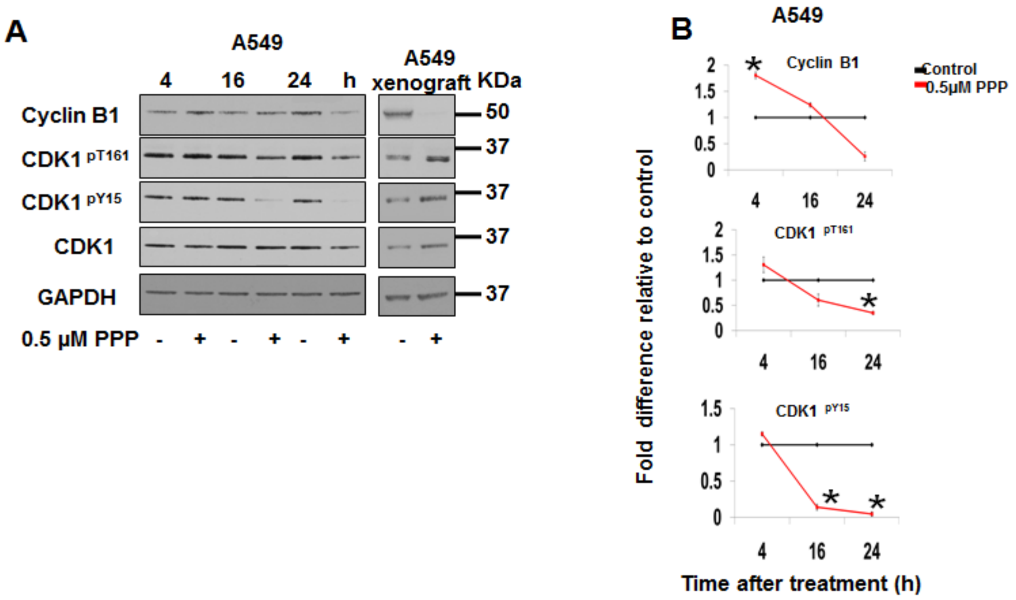

Supplementary figure 2: The effect of PPP on protein levels of cell cycle regulators in cultured A549 cells and in A549 xenografts. A549 cells were incubated with vehicle or with PPP for the indicated time points. Cell lysates were separated on 12% Bis-Tris SDS-PAGE and protein levels were detected by Western blot (15 µg protein loaded per lane). GAPDH served as a loading control. A549 xenografts were treated with PPP for 27 h or with vehicle; Cyclin B1 could not be detected in the xenografts (A). Densitometric analysis of the Western blots of A549 cells in culture (A) normalized to GAPDH are represented as fold-difference between the band intensities of PPP-treated cells (red) compared to vehicle-treated cells (black) (D).

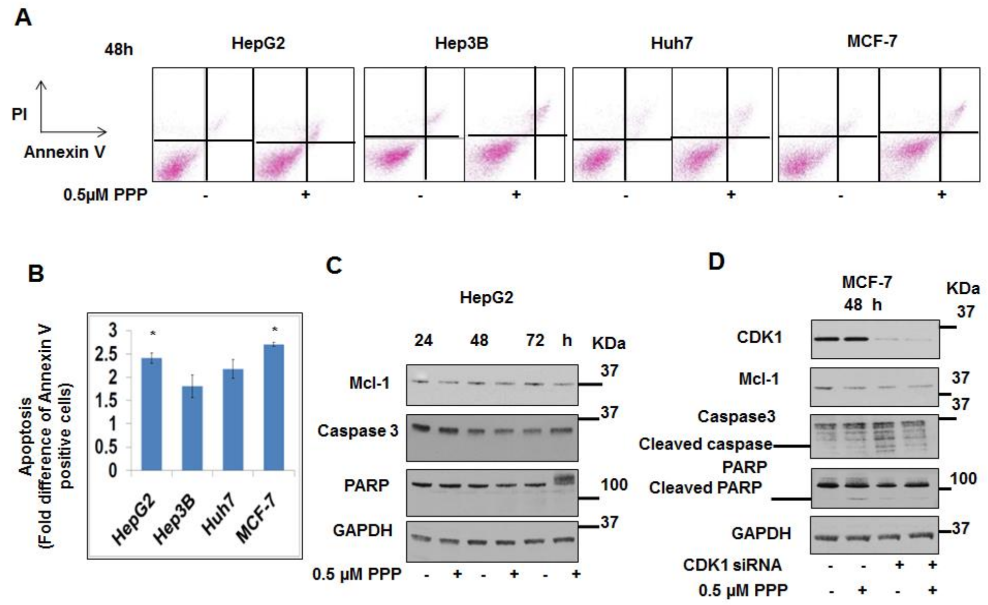

Supplementary figure 3: PPP induced cell death. A representative example of Annexin V/PI assay is shown in (A). Cancer cell lines were treated with 0.5 µM PPP for 48 h, stained with FITC-conjugated Annexin V/PI and subjected to flow cytometry. The lower left quadrant represents viable cells (AnV-/PI-), the lower right quadrant represents early apoptotic cells (AnV+/PI-ve), the upper right quadrant represents late apoptotic cells (AnV+/PI+) and the upper left quadrant represents necrotic cells (AnV-/PI+). The sum of FITC-positive cells in the right top and bottom quadrants representing the total percentage of early and late apoptotic cells is depicted in the histogram shown in (B). Western blot analysis of the levels of proteins regulating apoptosis from HepG2 cell lysates after treatment with PPP or with vehicle for the indicated time points (15 µg protein loaded per lane) (C). Western blot analysis of the levels of proteins regulating apoptosis from MCF-7 cell lysates after transfection with mock-siRNA or CDK1 siRNA for 72 h followed by PPP or vehicle treatment for 48 h (D). GAPDH served as a loading control in (C, D). PI: propidium iodide, FITC: fluorescein isothiocynate.

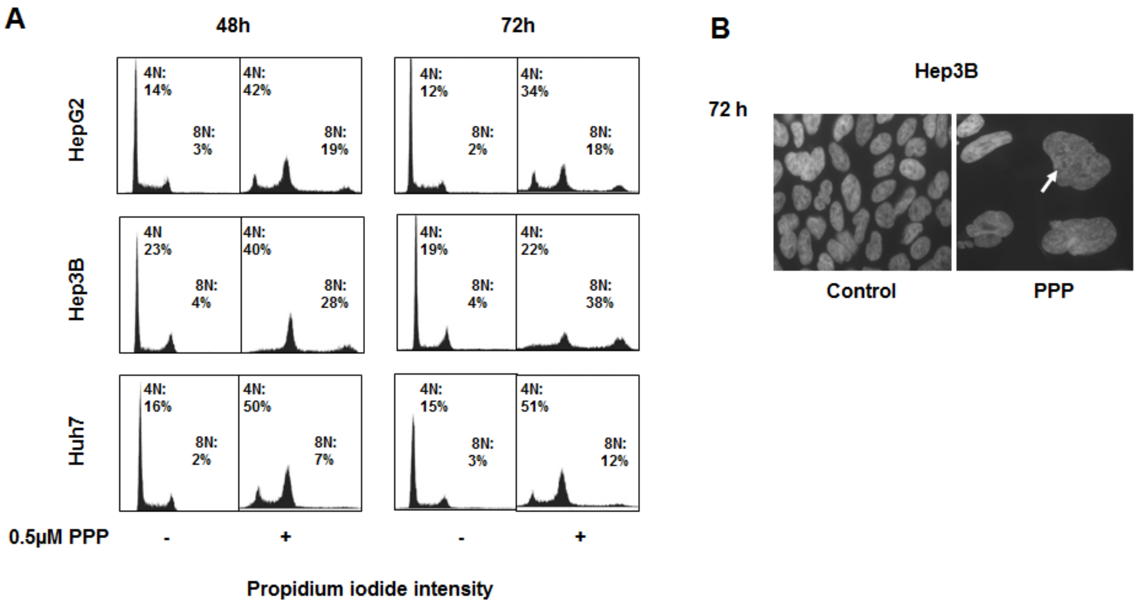

Supplementary figure 4: PPP induced polyploidy. FACS analysis of HepG2, Hep3B and Huh7 cell lines is shown after treatment with vehicle or with PPP for 48 and 72h (A). Hep3B cells were stained with DAPI after treatment with vehicle (left) or with PPP (right) and analyzed using immunofluorescence microscopy showing enlarged nuclei after PPP treatment (arrow) (B).

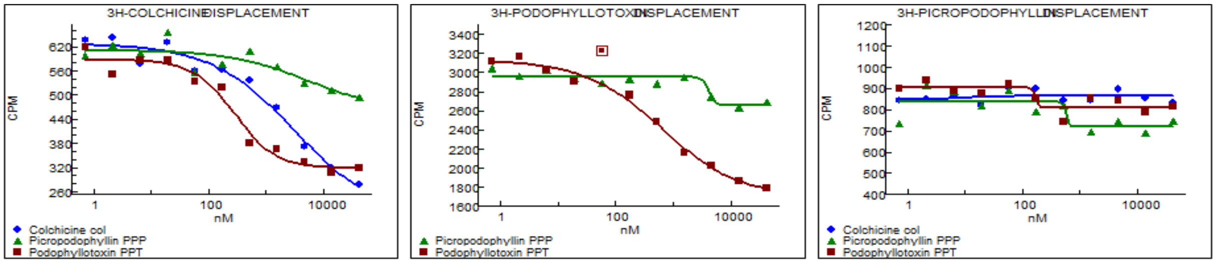

Supplementary figure 5: **Tubulin binding competition studies**. PPP, PPT and colchicine were prepared in increasing concentrations and the interference with tubulin binding of tritiated colchicine, PPT or PPP was investigated. Blue circle = colchicine, Green triangle = PPP, Red square = PPT. Data are presented as mean of duplicate samples.

Supplementary movie 1.
PPP prevented centrosome separation and establishment of a bipolar spindle during mitotic entry. Film shows maximum intensity projections of U2OS cells expressing mCherry-a-tubulin and GFP-Histone H2B after addition of DMSO (left) or PPP (right). Data refer to figures 4D and 5A and is shown at 3 min per frame.

Supplementary movie 2.
The FRET-ratio of a Plk1 responsive probe was not affected by PPP addition. Film shows false-colored inverted FRET ratio every 30 min. DMSO (left), PPP (middle), or BI2536 (right) were added after second frame. Data refer to figure 5C.

Supplementary movie 3.
PPP addition in metaphase led to spindle collapse. Mitotic cells were monitored 90 min after MG132 addition. PPP (left), Nocodazole (second left), STLC (second right), or DMSO (right) was added after first frame. Film shows maximum intensity projections of U2OS cells expressing mCherry-a-tubulin and GFP-Histone H2B at 9 min per frame. Data refer to figures 5B and 6.
